# Numerical Analyses of Blood Pumps Under Realistic Operating Conditions

**DOI:** 10.1097/MAT.0000000000002590

**Published:** 2025-11-04

**Authors:** Simon Klocker, Marko Grujic, Rosmarie Schöfbeck, Bente Thamsen, Benjamin Torner, Bernhard Semlitsch, Daniel Zimpfer, Marcus Granegger

**Affiliations:** From the * Christian Doppler Laboratory for Mechanical Circulatory Support, Department of Cardiac and Thoracic Aortic Surgery, Medical University of Vienna, Vienna, Austria; † Department of Fluid Flow Machinery, Technical University of Vienna, Vienna, Austria.

**Keywords:** rotodynamic blood pumps, ventricular assist device, realistic operating condition, pulsatile flow, computational fluid dynamics

## Abstract

Computational fluid dynamics (CFD) assessments in blood pumps (BPs) typically rely on constant boundary conditions, despite the dynamic nature of the cardiovascular system. Consensus on CFD methodologies for simulating BPs under realistic conditions is lacking, and qualitative validation against *in vitro* data, particularly regarding the dynamic pressure head-flow rate (HQ) hysteresis curve, remains absent. This study aims to validate a CFD framework capable of capturing HQ hysteresis. Time-varying boundary conditions were derived from a hybrid *in vitro* mock circulation. Computational fluid dynamics parameters, including boundary conditions (pressure *versus* mass flow), time step size (4°–72° per step), rotation modeling (frozen rotor *versus* sliding mesh), and turbulence modeling (none *versus k* − *ω* SST), were iteratively refined. Results were validated by assessing the overlap of simulated and measured HQ hysteresis using the Jaccard Index (JI). Dynamic HQ hysteresis was captured only with mass flow boundary conditions, not with pressure boundary conditions (JI = 0.62 *vs.* 0.37). Time step size, rotation modeling (except for frozen rotor without averaging), and turbulence modeling had minimal effect on HQ hysteresis but significantly influenced flow field resolution and computational efficiency. Critical parameters emerged in boundary conditions and motion modeling, whereas others involved trade-offs between flow field accuracy and computational cost.

## Graphical abstract

**Figure:**
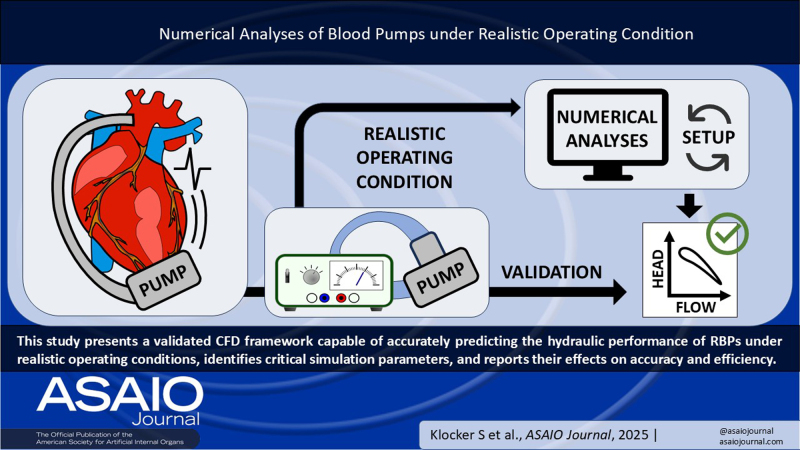

The use of rotodynamic blood pumps (RBPs) as left ventricular assist devices (LVADs) has become a standard therapy for patients with severe heart failure when donor organs are unavailable. Although these devices improve survival and quality of life, hemocompatibility-related adverse effects (HRAEs) remain a clinical burden.^1^ Hemocompatibility-related adverse effects, such as stroke, non-surgical bleeding, and thrombosis, are often linked to the damage, activation, and aggregation of blood components.

Computational fluid dynamics (CFD) can provide insights to understand and mitigate these effects. Typically, such CFD simulations use constant boundary conditions to compute flow fields, from which parameters like shear stress, exposure time, and stagnation zones are derived to estimate blood damage and thrombus formation.^2,3^

In the cardiovascular system, however, boundary conditions are not constant, and the native cardiac function, in combination with the pump’s hydraulic characteristics, results in pulsatile flow. Periodic flow changes during each cardiac cycle can lead to off-design operating conditions for over 50% of the cycle.^4^ These conditions are associated with compromised hemocompatibility, highlighting the need to analyze their effects to improve the RBP’s hemocompatibility under realistic operating conditions.^5,6^

In the pressure head-flow rate (HQ) diagram, dynamic boundary conditions result in a counterclockwise hysteresis loop around the static characteristic.^7^ The shape of the loop reflects the dynamic behavior of the system, which is attributed to the fluid inertia and the hydraulic properties of the pump—an effect already incorporated and validated in mathematical pump models.^4^

Although recent studies have started integrating realistic operating conditions into numerical assessments, as shown in Table 1, there is no consensus on which methodologies to employ.^8–16^ Moreover, qualitative *in vitro* validation that accounts for the shape of the hysteresis reflecting the dynamic system behavior is still lacking. Current validations typically rely either on comparisons with heuristically fitted mathematical models or on *in vitro* data obtained under static conditions.^8–16^

**Table 1.** Overview of Methodologies Used to Predict Dynamic Behavior in Previous Studies

Authors	Fluid	Boundary Conditions	Time Step	Physical Time	Rotation Modeling	Turbulence Modeling	Validation
Song *et al*.^8^	1,050 kg m^−3^ 3.5 mPa·s	LVQ, AOP	75°/TS	2 CC	Sliding mesh	k−ϵ	n.A.
Li *et al*.^9^	1,059 k m^−3^ 3.5 mPa·s	LVQ, AOP	33.6°/TS	n.A.	Sliding mesh	k−ω SST	*In vitro* with different fluid properties (1,100 kg m^−3^, 3.5 mPa·s)
Chen *et al*.^10^	1,050 kg m^−3^ 3.5 mPa·s	LVQ, AOP	n.A.	10 CC	Sliding mesh	k−ω SST	n.A.
Grinstein *et al*.^11^	1,060 kg m^−3^ 3.5 mPa·s	LVQ, AOP	n.A.	n.A.	n.A.	n.A.	n.A.
Huang *et al*.^12^	1,050 kg m^−3^ 3.5 mPa·s	LVP, AOP	18 °/TS	6 CC	Frozen rotor	k−ω SST	Static condition against *in vitro* results
Li *et al*.^13^	1,055 kg m^−3^ 3.5 mPa·s	LVP, AOP	96–108°/TS	2 CC	n.A.	k−ϵ	Static condition against *in vitro* results
Hahne *et al*.^14^	1,055 kg m^−3^ 3.5 mPa·s	LVP, AOP	18°/TS	3 CC	Sliding mesh	k−ω SST	Hydraulic model with different fluid properties (1,110 kg m^−3^, 3.0 mPa·s)
Wiegmann *et al*.^15^	1,050 kg m^−3^ 3.5 mPa·s	LVP, AOP	2°/TS	2 CC	Sliding mesh	k−ω SST	Hydraulic model with different fluid properties (1,110 kg m^−3^, 3.0 mPa·s)
Crone *et al*.^16^	1,050 kg m^−3^ 3.5 mPa·s	LVP, AOP	2°/TS	2 CC	Sliding mesh	k−ω SST	Hydraulic model with different fluid properties (1,110 kg m^−3^, 3.0 mPa·s)

AOP, pressure boundary condition at aortic side (outlet); CC, cardiac cycle; LVP, pressure boundary condition at left ventricular side (inlet); LVQ, flow boundary condition at left ventricular side (inlet); TS, time step.

The aim of this study is to validate a CFD framework capable of accurately and efficiently capturing the dynamic behavior of RBPs under realistic operating conditions.

## Materials and Methods

As the CFD framework was developed through an iterative process to achieve maximum accuracy, we first present its validation against *in vitro* data in its final form. This validated framework then serves as the benchmark in the analyses of simulation parameters aiming to investigate their effect on accuracy and computational efficiency, and to identify critical parameters.

All investigations were conducted using the HeartMate III (HM3, Abbott, Chicago, IL).

### *In Vitro* Validation

#### Experimental setup

**Figure 1.**
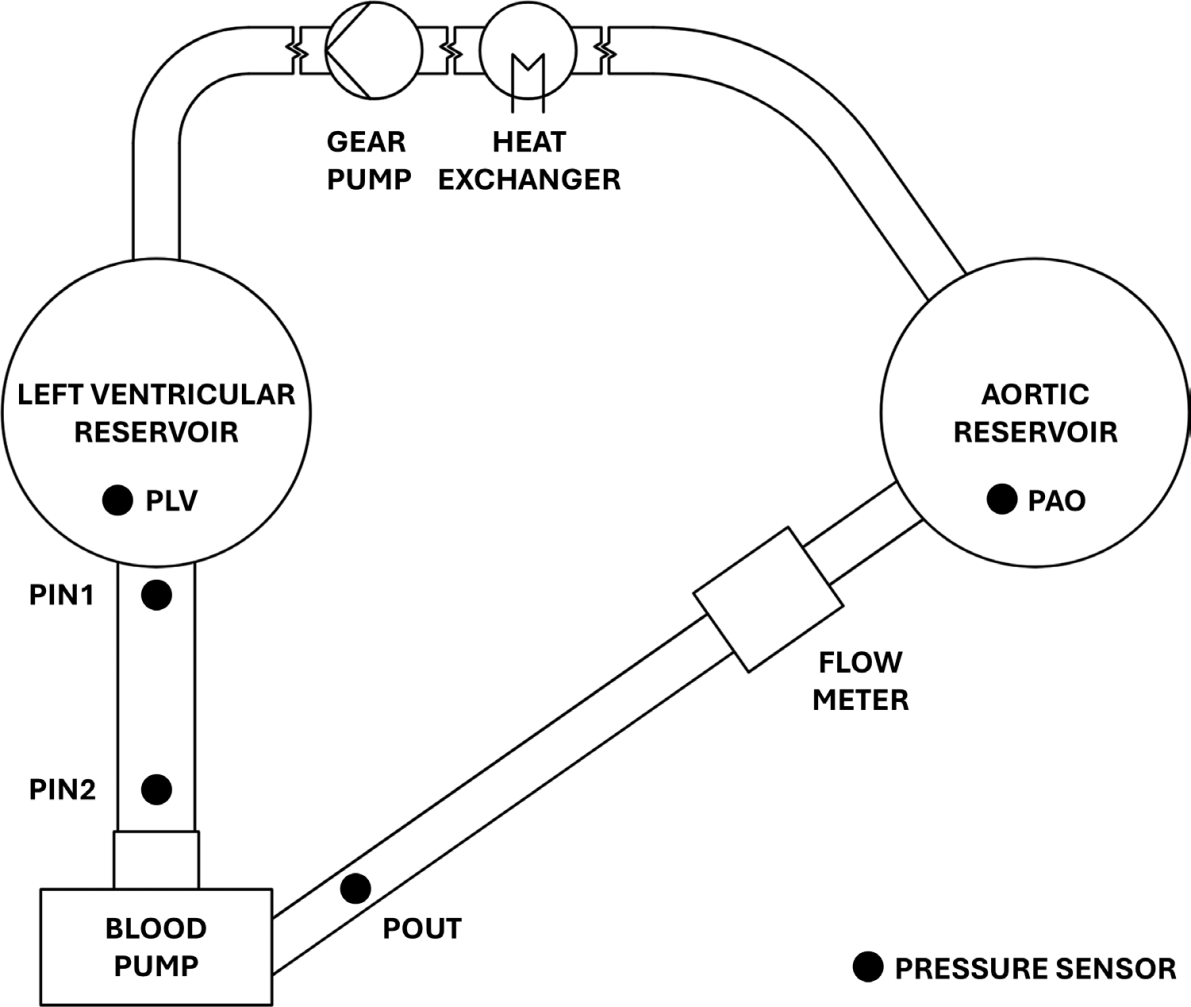
The mock loop setup consists of two pressure-controlled reservoirs with the RBP mounted in between. Additionally, a gear pump is used to maintain fluid levels in the reservoirs, and a heat exchanger is installed to maintain temperature-dependent fluid properties. Pressure is assessed in the reservoirs and at the pathways up and downstream of the RBP. RBP, rotodynamic blood pump.

Based on the design by Bender *et al*.,^17^ the experimental mock loop comprised two pressure-controlled reservoirs simulating the left ventricle and the aorta, with the RBP under investigation positioned between them, as illustrated in Figure 1. A separate gear pump (UP3-R 24V, MARCO s.p.a., Italy) was used to regulate the flow rate. An additional tube approximately 18 cm long was installed between the left ventricular reservoir and the RBP to mount additional pressure sensors monitoring the boundary conditions, subsequently used in the numerical setup. The outlet graft connecting the RPB and the aortic reservoir had a length of approximately 20 cm. Between the RBP and the aortic reservoir, a clamp-on ultrasonic flow meter (SONOFLOW CO.55, SONOTEC GmbH, Halle [Saale], Germany) measured the flow rate. Pressure sensors were positioned in the reservoirs (pressure in left ventricular reservoir [PLV]; pressure in aortic reservoir [PAO]), as well as at specific points along the inflow and outflow pathways of the RBP, see Figure 1. Sensor PIN1 was placed 150 mm upstream, PIN2 was positioned 10 mm upstream of the cannula inlet, and sensor POUT was located 30 mm downstream of the outlet duct.

All experiments were conducted using a water-glycerol mixture (50.7% glycerol by weight) with a dynamic viscosity of 3.5 mPa·s and a density of 1,120 kg/m³ at 37°C. A heat exchanger is placed downstream of the aortic reservoir to maintain a constant temperature of 37°C. Fluid properties were verified by measuring the refractive index with a refractometer at 21°C to determine the corresponding glycerol mass percentage.^18^ Dynamic viscosity and density were determined using a density model that accounts for temperature effects.^19,20^

#### Hydraulic assessment

In static operation, the gear pump was controlled to deliver the desired flow rate, whereas the rotational speed of the RBP was adjusted to achieve the requested pressure head. Static hydraulic characteristics of the RBP were assessed at various pump speed settings by gradually increasing the flow rate generated by the gear pump until the pressure head became negative. The resulting pressure head was calculated as the difference between the pressure measured at the sensor positioned downstream of the outlet duct (POUT) and upstream of the inlet cannula (PIN2). Due to the differing diameters of the inlet and outlet tubes, the dynamic pressure was added during post-processing to obtain the total pressure.

In dynamic operation, the reservoir pressures were controlled to replicate a virtual patient’s condition provided by a lumped parameter model, whereas the gear pump maintained consistent fluid levels in the reservoirs.^17,21^ The mock loop simulated an LVAD patient with partial support, meaning that the heart still contributes to the cardiac output (aortic valve opens). This scenario results in the highest amount of dynamic behavior. The cardiovascular model parameters, as previously reported, have been adopted.^17^ The waveforms of the left ventricular and aortic pressures, as well as the mass flow rate, are provided in Supplemental Digital Content, https://links.lww.com/ASAIO/B681.

A time series capturing approximately 40 cardiac cycles was recorded to assess dynamic behavior. The data were divided into individual cardiac cycles and subsequently averaged across all cycles to derive a representative measurement of the virtual patient’s pressures and flow rate. Notably, the Artificial Pulse feature of the HM3 was deactivated, allowing for analysis solely of the inherent dynamic behavior induced by the cardiac cycle.

#### Numerical setup

For the numerical flow investigation, the software Simcenter STAR-CCM+ (Siemens Digital Industries Software, Plano, Texas, USA) was used. The HM3 geometry was reconstructed from a high-resolution computed tomography (CT) scan.^6^ The geometry was adjusted to align with the pressure sensor positions in the mock loop to apply pressure boundary conditions. As a result, the inlet was extended by 150 mm and the outlet by 30 mm.

For spatial discretization, a polyhedral grid was used for the pump volume, whereas structured grids were employed to extend the inlet and outlet regions. Additionally, 10 prism layers were added along the walls to improve boundary layer resolution. The mesh quality criteria recommended by STAR-CCM+ had been satisfied, with all cells exhibiting a volume change greater than 0.01, skewness angles below 85°, and an average cell aspect ratio of 0.83 on a scale from 0 to 1, where a value of 1 represents a regular polygon.^22^ The y+ values reached a maximum of 1 at the tips of the blades. The total mesh consisted of 13.9 million cells. Mesh independence was confirmed using the procedure outlined by ASME, and results are reported in Supplemental Digital Content, https://links.lww.com/ASAIO/B681.^23^

**Figure 2.**
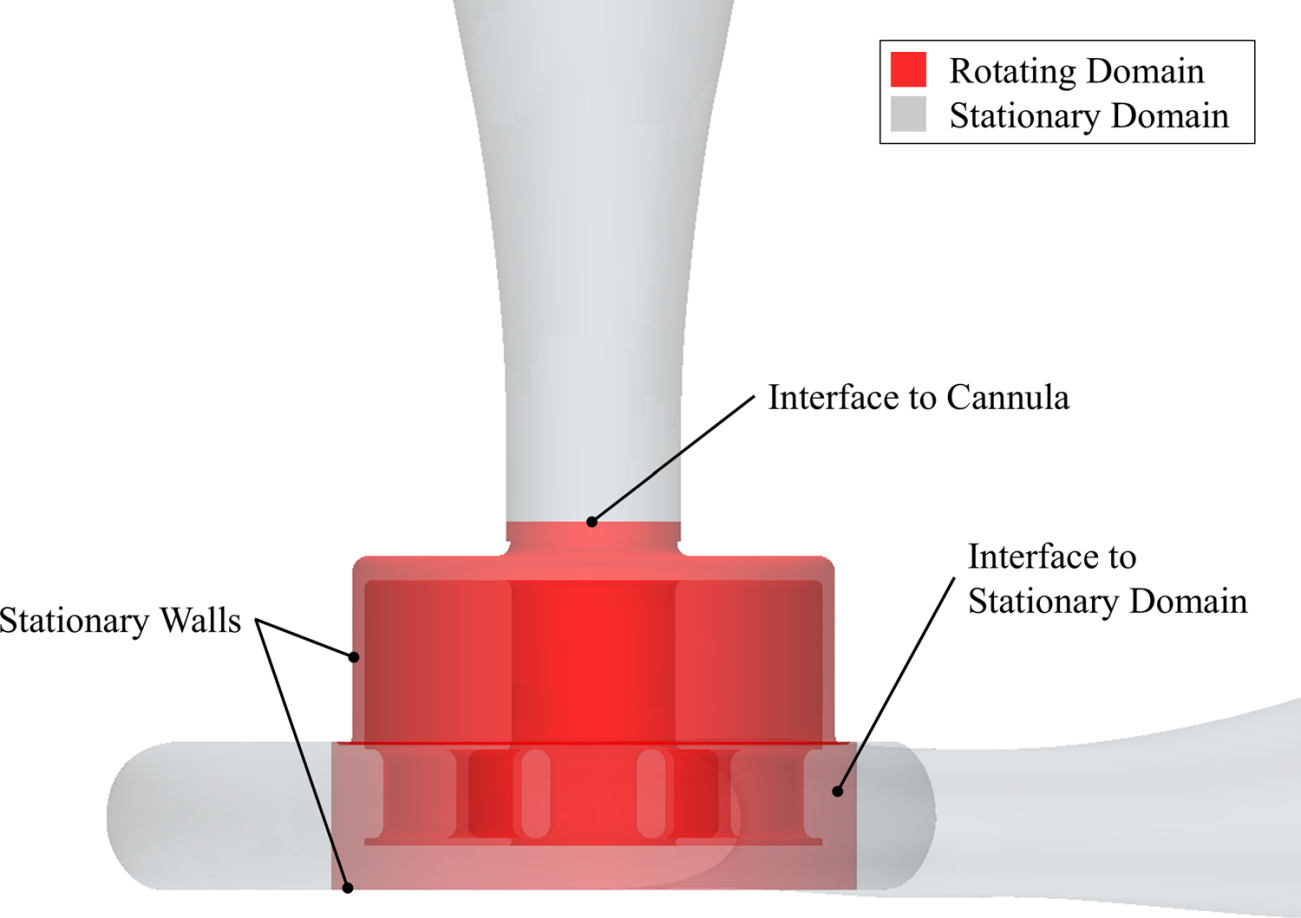
The rotating domain is confined by a cylinder 1.4 mm distant from the impeller walls and the cannula. The side gaps are included; therefore, the housing walls are defined as stationary walls.

As illustrated in Figure 2, the rotating domain included the fluid within and around the levitated impeller, including the gaps between the impeller and the housing. It was confined by the interface at the cannula towards the inlet and by a cylindrical interface located 1.4 mm from the outer impeller walls.

The governing equations were solved using a segregated flow approach, with spatial discretization achieved through a second-order upwind scheme and second-order temporal discretization.

Simulations were performed using Newtonian fluid properties consistent with the experimental conditions (density of 1,120 kg/m³, dynamic viscosity of 3.5 mPa·s). The assumption of Newtonian behavior was subsequently validated by showing that 91% of the volume within the pump, or 71% when including the cannula, was exposed to shear rates exceeding 100 s−¹, a commonly used threshold for assuming Newtonian behavior.^8–10,12,14,16^

Residuals decreased to below 10^−4^ at each time step to ensure numerical convergence, whereas physical convergence was achieved after simulating two overlapping cardiac cycles.

Simulation parameters—including boundary conditions, time step size, rotation modeling, and turbulence modeling—were selected through an iterative process to maximize accuracy. The final simulation setup employs time-varying mass flow boundary conditions, 4° rotation per time step, and a sliding mesh. Turbulence is considered using an unsteady Reynolds-averaged Navier–Stokes (URANS) approach utilizing *k* − ω SST turbulence modeling.^24^ This setup serves as the benchmark for the subsequent analyses of simulation parameters.

#### Dynamic quantification

The system’s dynamic behavior is reflected in hysteresis in the HQ diagram. To evaluate the method’s ability to predict dynamic behavior, the Jaccard Index (JI) between the measured and simulated hysteresis,


$$JI=\frac{Area\;of\;Overlap}{Area\;of\;Union},$$ (1)

was determined.^25^ Thus, the enclosed area and the hysteresis shape were considered. The same quantification was used by Crone *et al*.,^16^ where it was referred to as the overlapping ratio (OR).

### Analyses of simulation parameters

The analysis of simulation parameters builds upon the benchmark setup. The same mesh, discretization methods, and convergence criteria were applied.

The accuracy of the results obtained from various simulation parameters was quantified using the root mean square error (RMSE) of the predicted pressure head relative to the benchmark.

Although the accuracy of the predicted results is a prerequisite, computational cost is another factor to consider when selecting the appropriate methodology. Therefore, we report the elapsed CPU (central processing unit) solver time obtained from STAR-CCM+, normalized relative to the benchmark solution.

#### Time-varying boundary condition

Both approaches used in previous literature to define time-varying boundary conditions were compared: one with pulsatile mass flow at the inlet and a set reference pressure at the outlet (benchmark),^8–11^ and the other with pulsatile static pressure conditions at both the inlet and outlet.^12–16^ In both cases, the results from *in vitro* measurements were applied as time-varying boundary conditions.

When applying pressure boundary conditions, the simulation predicts mass flow, preventing a direct comparison with the pressure head from the benchmark setup. Therefore, the JI of both approaches was evaluated against the *in vitro* results.

#### Rotation modeling

Three approaches were used to model the rotational motion: sliding mesh (benchmark), frozen rotor in two positional configurations, and frozen rotor with mixing plane interface.^26^ In the sliding mesh approach, the impeller position is updated at each time step, whereas in the frozen rotor method, the impeller position remains fixed. Consequently, the rotor-stator interaction is not captured in the frozen rotor method, potentially leading to unphysical flow phenomena. The mixing plane interface circumferentially averages the flow between the rotating and stationary domains to address these phenomena.

#### Time step size

Time steps corresponding to rotations ranging from 4° (benchmark) to 16° for the sliding mesh and from 4° to 72° for the frozen rotor with a mixing plane interface were investigated. For the sliding mesh, the time step was not increased further to resolve the pressure fluctuations caused by rotor-stator interaction. Because the same state occurs every 90° for a four-blade impeller, a time step corresponding to 16° rotation provides five sample points per state, satisfying the Nyquist–Shannon theorem.^27^ This constraint does not apply to frozen rotor simulations.

#### Turbulence modeling

The governing equations were solved by addressing the Navier–Stokes equations both without a turbulence model and using the URANS approach, utilizing the *k − ω* SST turbulence model (benchmark). This enabled the analysis of whether different simulation methods with distinct turbulence modeling approaches can accurately capture the HQ hysteresis.

## Results

### *In Vitro* Validation

**Figure 3.**
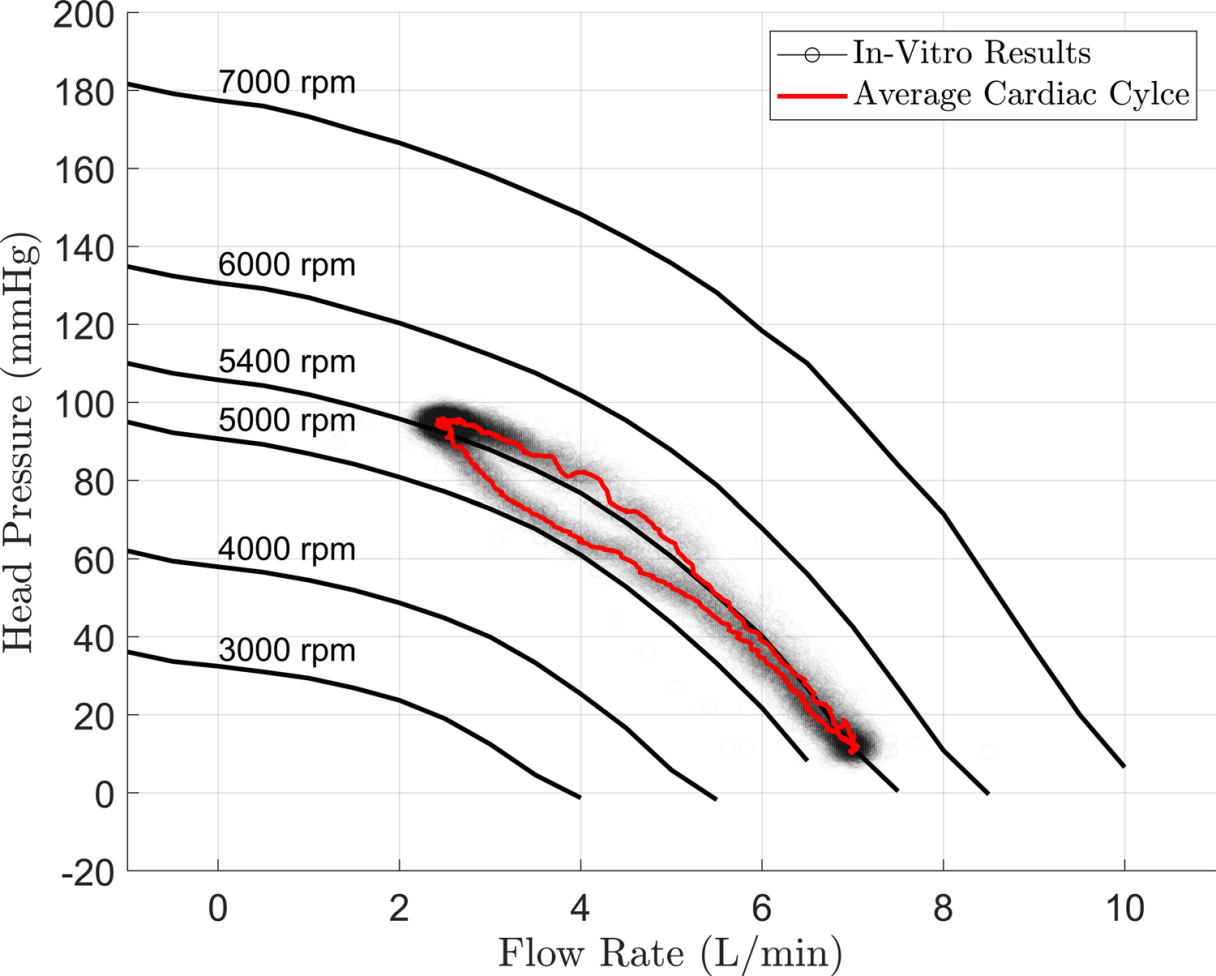
*In vitro* results showing the static characteristics at various pump speeds and the dynamic behavior of a virtual patient’s cardiac cycle.

Figure 3 presents the *in vitro* measurements of both static and dynamic conditions, highlighting the average cardiac cycle in red. The mean value and standard deviation for measured pressure head and flow rate are outlined in Supplemental Digital Content, https://links.lww.com/ASAIO/B681.

**Figure 4.**
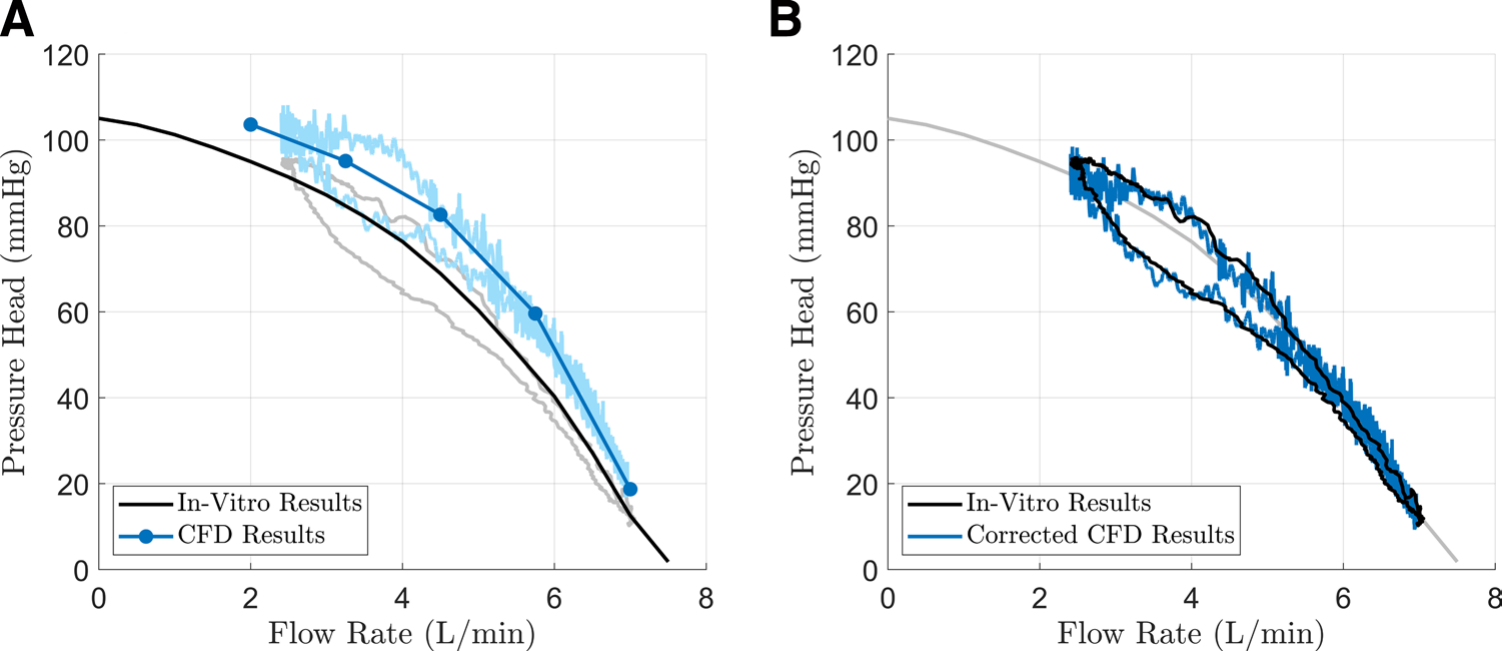
Comparison between numerical and in vitro results. **A**: In static operation, numerical results overpredict *in vitro* measurements. **B**: Comparison of the pressure head versus flow rate diagram’s hysteresis from *in vitro* experiments and the proposed simulation. After applying static correction, the simulation results closely align with experimental validation (root mean square error = 3.8 mm Hg, Jaccard Index = 0.62). CFD, computational fluid dynamics.

The numerically calculated pressure head overpredicted the *in vitro* results, as shown in Figure 4B. A similar overprediction was observed under static operation, as illustrated in Figure 4A, for five representative flow rates (2, 3.25, 4.5, 5.75, and 7 L/min), covering the entire dynamic operating range.

To assess the numerical setup’s ability to capture the system’s dynamic behavior the numerically calculated pressure head was corrected for static overprediction by aligning it with the measured characteristic line.

After applying this correction, the simulation results aligned strongly with the validation experiment (RMSE = 3.8 mm Hg, JI = 0.62). With time-averaged results, excluding fluctuations from rotor-stator interaction, the JI increased to 0.67.

### Analyses of Simulation Parameters

#### Time-varying boundary conditions

**Figure 5.**
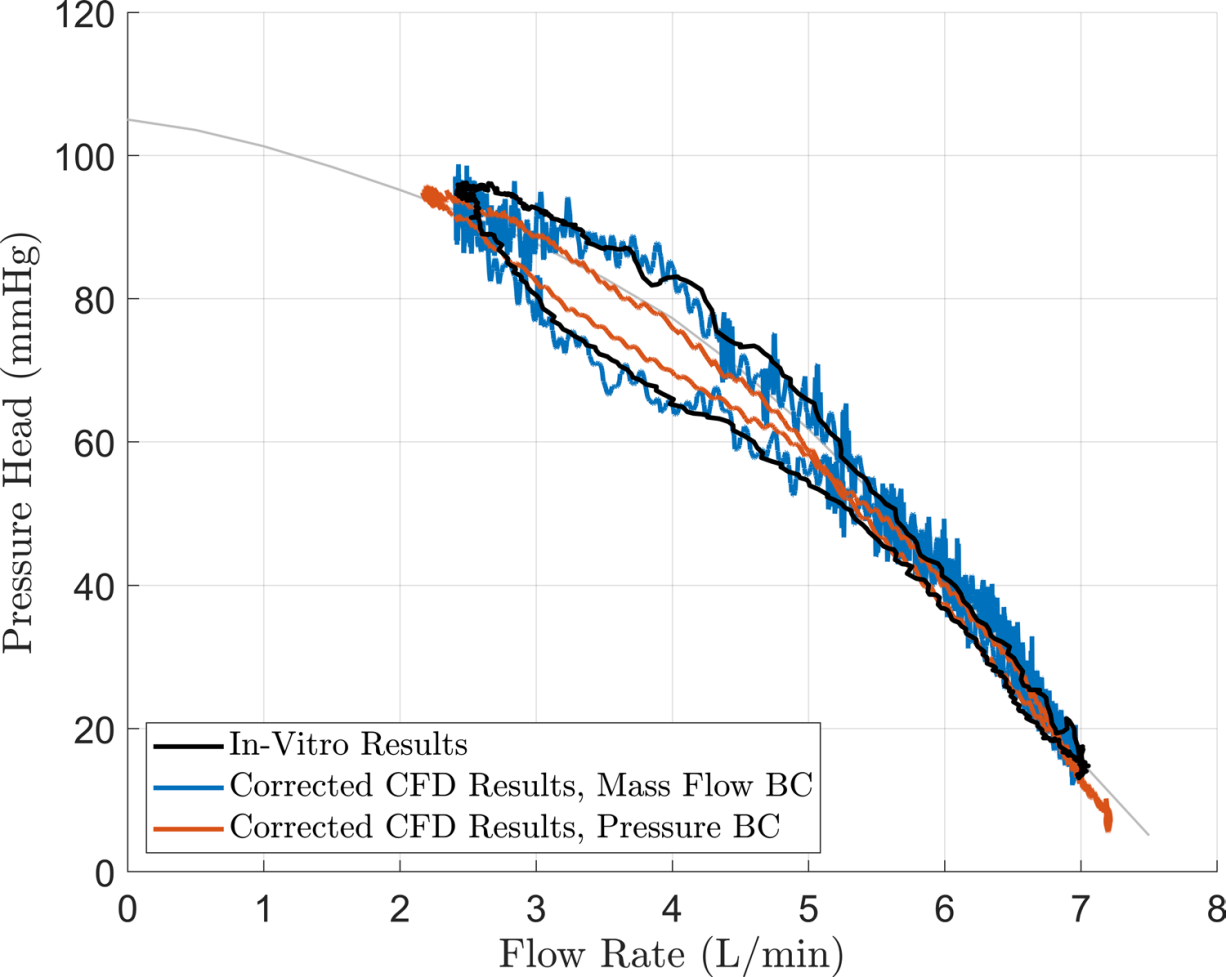
The predicted hysteresis using time-varying mass flow boundary conditions and time-varying pressure boundary conditions is compared against *in vitro* measurements. Both predicted hystereses are corrected for static overprediction. Although the dynamic behavior is captured with the mass flow boundary condition (Jaccard Index = 0.62), the pressure boundary conditions result in a narrowing of the hysteresis (Jaccard Index = 0.37). BC, boundary condition; CFD, computational fluid dynamics.

Both methods for defining time-varying boundary conditions, pulsatile mass flow rate, and pulsatile pressure were evaluated (Figure 5). Numerical results were corrected for static overprediction. The JI for the benchmark using time-varying boundary conditions was 0.62. In contrast, applying a time-varying pressure boundary condition resulted in a significantly lower JI of 0.37.

#### Rotation modeling

**Figure 6.**
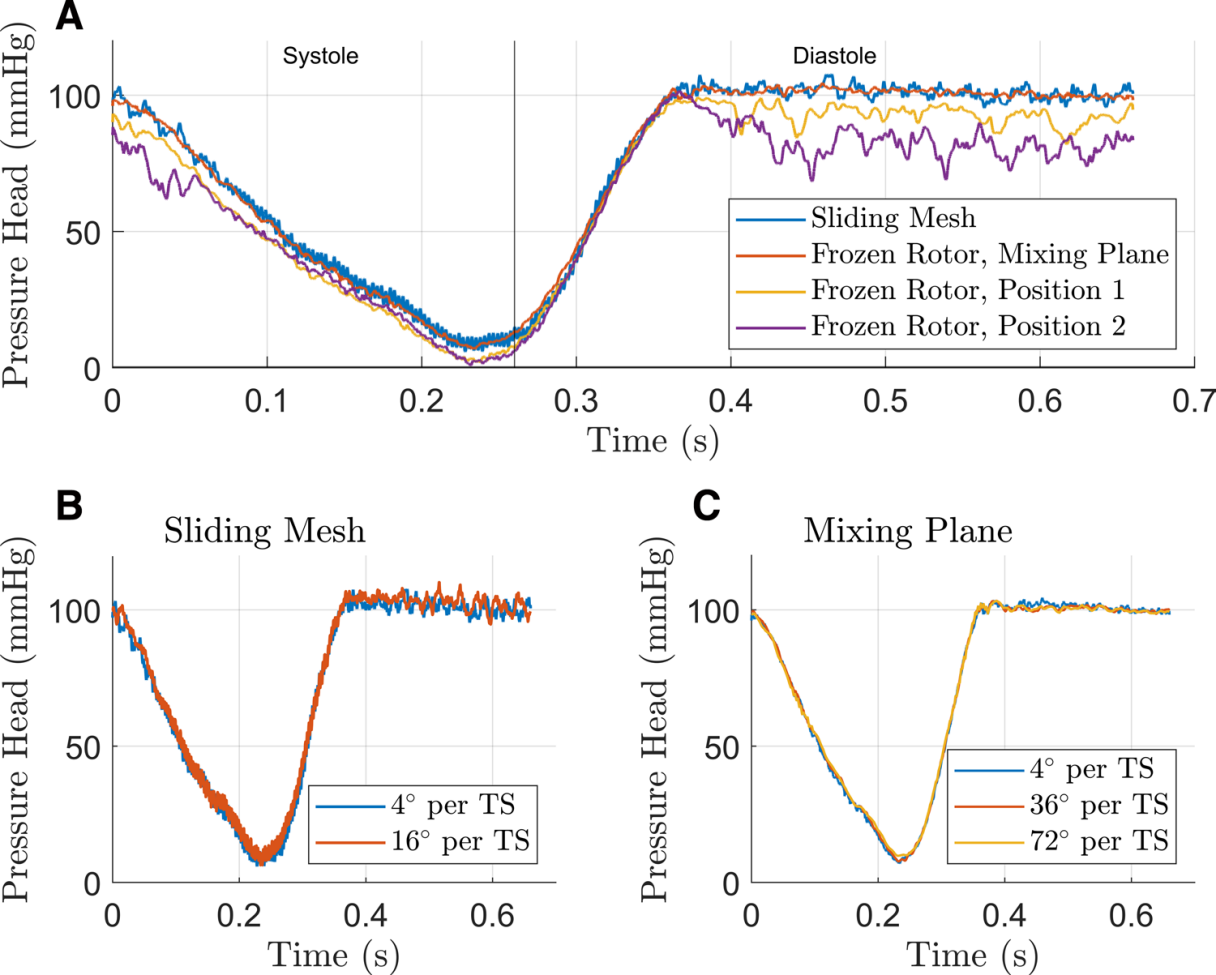
Numerical analyses of rotation modeling and time step size. **A**: The predicted pressure head is evaluated using various rotation modeling methods. The frozen rotor approach fails to accurately predict pressure head, whereas the frozen rotor with the mixing plane interface results aligns closely with the sliding mesh results. **B, C**: Results utilizing different time step sizes for the sliding mesh and frozen rotor with mixing plane interface remain closely aligned.

The dynamic HQ curves were predicted using sliding mesh, frozen rotor in two positional configurations, and frozen rotor with a mixing plane interface (Figure 6A). The frozen rotor method with fixed positions failed to accurately predict the pressure head (RMSE = 7.5 mm Hg, 14 mm Hg). In contrast, the frozen rotor with a mixing plane interface closely aligned with the sliding mesh results (RMSE = 2.7 mm Hg).

#### Time step size

A time step study was conducted for the sliding mesh (4° and 16° rotation per time step) and frozen rotor with the mixing plane interface (4°–72° rotation per time step), as presented in Figure 6, B and C.

Results with increased time steps remained well-aligned with the benchmark solution for both setups (sliding mesh with 16°: RMSE = 2.9 mm Hg, frozen rotor with mixing plane with 72°: RMSE = 2.6 mm Hg).

#### Simulation method and turbulence modeling

**Figure 7.**
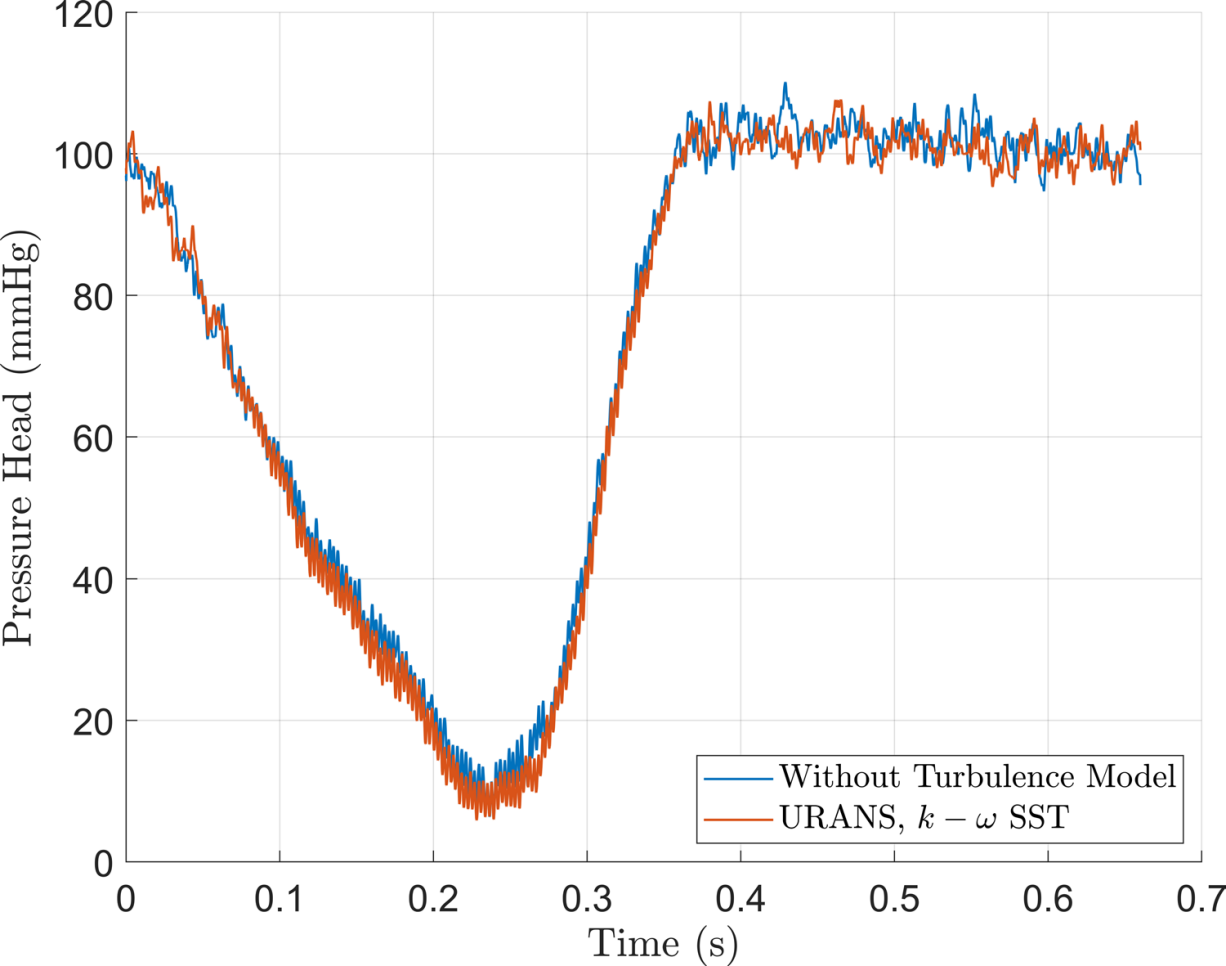
The predicted pressure head solving the governing equations without a turbulence model and URANS equations including the *k* − ω SST turbulence model remain comparable (root mean square error = 1.66 mm Hg). URANS, Unsteady Reynolds-Averaged Navier–Stokes.

The results obtained from solving the governing equation in the absence and presence of turbulence modeling are compared in Figure 7. The predicted pressure heads showed a deviation of less than 3 mm Hg, demonstrating the overall agreement between the two approaches, with an RMSE of 1.66 mm Hg.

#### Numerical efficiency assessment

The normalized elapsed CPU solver time was calculated for the smallest and largest time steps used in both rotation modeling approaches. A significant reduction in elapsed CPU solver time was observed for larger time steps in both approaches: −29% for the sliding mesh approach (16° rotation per time step) and −90% for the frozen rotor approach with a mixing plane (72° rotation per time step). However, solely averaging at the mixing plane interface by remaining the same time step increased the elapsed CPU solver time by 56%. One cardiac cycle required 1.0 × 10⁴ core hours using the benchmark setup.

## Discussion

### *In Vitro* Validation

We demonstrated that by integrating time-varying mass flow boundary conditions, a 4° rotation per time step, a sliding mesh, and a URANS approach with *k* − *ω* SST turbulence modeling, the dynamic behavior was accurately captured. To evaluate this, we calculated the JI and validated it against *in vitro* data. This qualitative validation using dynamic experimental data contrasts with earlier studies that relied on static conditions or heuristically fitted models with inconsistent fluid properties.^8–16^ To our knowledge, only Li *et al.*^9^ performed pulsatile experiments for validation. However, their setup featured inconsistent fluid density, despite maintaining constant viscosity, and qualitative assessment is lacking. Although Crone e*t al.*^16^ also applied the JI, their validation was limited to comparisons with a heuristically fitted model.

Of note, validation against *in vitro* measurements required the adjustment of the static overprediction of the pressure head. This overprediction may stem from a combination of factors, including deviations between the experimentally and numerically assessed HM3, potential wall roughness effects, and oscillating impeller movement.

This correction is required because the JI accounts for both the overlap between hysteresis loops and the penalty from any residual area. Consequently, shifts in the relative position of the enclosed hysteresis loops distort the JI.

In contrast to our findings, previous studies have reported strong agreement between *in vitro* measurements and CFD results. This discrepancy may be attributed to differences in density values (Table 1) between the blood analogs (1,110 kg/m³) used for validation and those in simulations (1,050–1,055 kg/m³). A density difference of approximately 6% is expected to increase the pressure head proportionally.^28^

Although water-glycerol solutions are commonly used as blood analogs, their density is higher than that of blood when adjusted to match its viscosity. Knüppel *et al.*^28^ suggested the blood analog PEG200, which allows matching both viscosity and density of blood.

Because this correction is not applicable to the local flow field, investigations based on the resolution of the flow, such as shear stress and blood damage, remain limited.

### Analyses of Simulation Parameters

#### Time-varying boundary condition

The finding that the use of a time-varying mass flow boundary condition is necessary to accurately capture dynamic behavior contrasts with previous studies, which used time-varying pressure boundary conditions at the inlet and outlet.^12–16^

Unlike mass flow boundary conditions, which are placement-independent due to conservation laws, pulsatile pressure boundary conditions require precise positioning. In this study, the boundaries were chosen to replicate the locations of the pressure sensors in the mock circulation. However, the pressure fluctuations caused by rotor-stator interaction were less pronounced when using pressure boundary conditions, indicating that the boundaries were positioned too close and constrained the pressure field in the region of interest.

This lack of dependency on mass flow conditions simplifies the validation and eliminates the need for an additional pipe between the left ventricular reservoir and the RBP, as pressure sensors at specific locations are not required to match the CFD setup. Moreover, removing the additional pipe allows for direct suction from the reservoir, which more closely aligns with clinical applications.

Additionally, whereas the results in our study using time-varying mass flow boundary conditions showed negligible dependency on time step size, Crone *et al.*^16^ reported a significant time step dependency of the predicted HQ diagram’s hysteresis. Notably, in their setup, pressure boundary conditions were imposed through a lumped parameter model, which is coupled with the CFD solver at each time step.

#### Rotation modeling and time step size

Prior studies showed consensus, with one exception, on the use of sliding meshes to model impeller rotation.^12^ This choice is supported by the validation conducted in this study. Additionally, resolving transient local flow fields, achievable only with a sliding mesh, is required for predicting blood damage, making it the preferred method.

The frozen rotor method predicted hydraulic performance less accurately and exhibited a dependency on the applied position. This discrepancy was particularly evident during diastole, as illustrated in Figure 6A, a phase characterized by partial load pump operation and pronounced pressure fluctuations caused by transient flow phenomena (flow separations, large-scale eddies, and recirculation). Consequently, the limitations of the frozen rotor approach render it less suitable for capturing the dynamics of transient flow.

We demonstrated that using a mixing plane interface with the frozen rotor accurately captured dynamic behavior. This approach remains valid even with increased time step sizes, enabling substantial computational cost reductions. Yet, this gain in efficiency comes at the expense of transient flow field resolution due to circumferential averaging at the interface. Therefore, mixing plane interfaces are beneficial primarily when hydraulic performance is the focus.

#### Simulation method and turbulence modeling

Turbulence generates additional shear stress on blood cells, contributing to blood damage.^29,30^ Therefore, incorporating turbulence is essential. In RBPs, turbulence can be expected due to interactions between secondary flows (*eg*, leakage flows, flow separations, and boundary layers) and the primary flow path, leading to shear layers and turbulence production.^31^

We observed that simulations without a turbulence model performed similarly in terms of dynamic pressure head compared to URANS computations using the *k* − *ω* SST turbulence model. Although this may seem counterintuitive, a possible explanation is that the absence of a turbulence model allows the simulation to directly resolve a portion of the turbulence. This is likely due to our simulation setup’s fine discretization in time and space.

Crone *et al.*^16^ performed a power loss analysis on the same pump using a turbulence-resolving large eddy simulation (LES). Their study found that the hydraulic performance was accurately predicted with an 11-million-cell mesh compared to a 70-million-cell mesh. Contrarily, the fluidic shear stress was not accurately captured.

This aligns with our findings that the hydraulic performance can be reliably estimated with turbulence-resolving simulations on a comparable mesh. However, this approach has yet to be validated for fluid shear stress and blood damage predictions.

Moving forward in this direction, a shear stress formulation that accounts for stresses from both the resolved and modeled components should be considered.^32^ Such a formulation is applicable across various simulation methods, including URANS, LES, and direct numerical simulation. A logical next step would be to analyze these stresses and the associated blood damage under realistic operating conditions.

## Conclusions

Our findings demonstrate that CFD simulations can accurately capture hydraulic properties under realistic operating conditions, with time-varying mass flow boundary conditions identified as a critical simulation parameter. The setup was validated by comparing both the deviation and shape of the predicted *versus* measured HQ hysteresis, reflecting the system’s dynamic behavior.

Additionally, analysis of simulation parameters provided valuable insights into both accuracy and efficiency, leading to the following recommendations:

Global assessments, such as hydraulic performance, can be reliably predicted using larger time steps and mixing plane interfaces within the frozen rotor approach. This enables significant reductions in computational costs, albeit at the expense of local flow resolution. In contrast, analyses focusing on local flow phenomena, such as blood damage prediction, require the use of a sliding mesh.

## Acknowledgments

The computational results have been achieved using the Austrian Scientific Computing (ASC) infrastructure.

## Supplementary Material

## References

[1] Meyer DM Nayak A Wood KL: The Society of Thoracic Surgeons Intermacs 2024 Annual Report: Focus on outcomes in younger patients. Ann Thorac Surg 119: 34–58, 2025. 39442906 10.1016/j.athoracsur.2024.10.003

[2] Garon A Farinas M-I: Fast Three-dimensional numerical hemolysis approximation. Artif Organs 28: 1016–1025, 2004. 15504117 10.1111/j.1525-1594.2004.00026.x

[3] Wu W-T Yang F Wu J Aubry N Massoudi M Antaki JF: High fidelity computational simulation of thrombus formation in Thoratec HeartMate II continuous flow ventricular assist device. Sci Rep 6: 38025, 2016. 27905492 10.1038/srep38025 PMC5131309

[4] Boes S Thamsen B Haas M Daners MS Meboldt M Granegger M: Hydraulic characterization of implantable rotary blood pumps. IEEE Trans Biomed Eng 66: 1618–1627, 2019. 30334747 10.1109/TBME.2018.2876840

[5] Escher A Hubmann EJ Karner B: Linking hydraulic properties to hemolytic performance of rotodynamic blood pumps. Advcd Theory Sims 5: 2200117, 2022.

[6] Schöps M Groß-Hardt SH Schmitz-Rode T: Hemolysis at low blood flow rates: In-vitro and in-silico evaluation of a centrifugal blood pump. J Transl Med 19: 2, 2021. 33402176 10.1186/s12967-020-02599-z PMC7784380

[7] Stanfield JR Selzman CH: In vitro pulsatility analysis of axial-flow and centrifugal-flow left ventricular assist devices. J Biomech Eng 135: 0345051–0345056, 2013. 10.1115/1.4023525 PMC3705790 24231821

[8] Song X Throckmorton AL Wood HG Allaire PE Olsen DB: Transient and quasi-steady computational fluid dynamics study of a left ventricular assist device. ASAIO J 50: 410–417, 2004. 15497378 10.1097/01.mat.0000136507.57707.0f

[9] Li H Gou Z Huang F Ruan X Qian W Fu X: Evaluation of the hemolysis and fluid dynamics of a ventricular assist device under the pulsatile flow condition. J Hydrodyn 31: 965–975, 2019.

[10] Chen Z Jena SK Giridharan GA: Flow features and device‐induced blood trauma in CF‐VADs under a pulsatile blood flow condition: A CFD comparative study. Numer Methods Biomed Eng 34: e2924, 2018. 10.1002/cnm.2924 PMC5803368 28859253

[11] Grinstein J Torii R Bourantas CV Garcia-Garcia HM: Left ventricular assist device flow pattern analysis using a novel model incorporating left ventricular pulsatility. ASAIO J 67: 724–732, 2021. 33528162 10.1097/MAT.0000000000001341

[12] Huang F Lei H Ying S Fu Y Li Q Ruan X: Numerical hemolysis performance evaluation of a rotary blood pump under different speed modulation profiles. Front Physiol 14: 1116266, 2023. 36818439 10.3389/fphys.2023.1116266 PMC9931726

[13] Li S Jin D Gui X: Dynamic characteristic modeling of left ventricular assist devices based on hysteresis effects. Comput Biol Med 157: 106737, 2023. 36921456 10.1016/j.compbiomed.2023.106737

[14] Hahne M Crone V Thomas I: Interaction of a ventricular assist device with patient-specific cardiovascular systems: In-Silico study with bidirectional coupling. ASAIO J 70: 832–840, 2024. 38551498 10.1097/MAT.0000000000002181 PMC11426988

[15] Wiegmann L Thamsen B De Zélicourt D: Fluid dynamics in the HeartMate 3: Influence of the artificial pulse feature and residual cardiac pulsation. Artif Organs 43: 363–376, 2019. 30129977 10.1111/aor.13346

[16] Crone V Hahne M Knüppel F Wurm F-H Torner B: Dynamic VAD simulations: Performing accurate simulations of ventricular assist devices in interaction with the cardiovascular system. Int J Artif Organs 47: 624–632, 2024. 39238170 10.1177/03913988241268067 PMC11656629

[17] Bender M Escher A Messner B: An atraumatic mock loop for realistic hemocompatibility assessment of blood pumps. IEEE Trans Biomed Eng 71: 1651–1662, 2024. 38133971 10.1109/TBME.2023.3346206

[18] Hoyt LF: New table of the refractive index of pure glycerol at 20°C. Ind Eng Chem 26: 329–332, 1934.

[19] Cheng N-S: Formula for the viscosity of a glycerol−water mixture. Ind Eng Chem Res 47: 3285–3288, 2008.

[20] Volk A Kähler CJ: Density model for aqueous glycerol solutions. Exp Fluids 59: 75, 2018.

[21] Colacino FM Moscato F Piedimonte F Danieli G Nicosia S Arabia M: A modified elastance model to control mock ventricles in real-time: Numerical and experimental validation. ASAIO J 54: 563–573, 2008. 19033767 10.1097/MAT.0b013e31818a5c93

[22] Siemens Digital Industries Software: Simcenter STAR-CCM+ User Guide 2310, Siemens, 2023. Available at: https://docs.sw.siemens.com/en-US/doc/226870983/PL20230724207774020.starccmp_userguide_html?audience=external.

[23] Celik IB Ghia U Roache PJ Freitas CJ Coleman H Raad PE: Procedure for estimation and reporting of uncertainty due to discretization in CFD applications. J Fluids Eng 130: 078001, 2008.

[24] Menter FR: Two-equation eddy-viscosity turbulence models for engineering applications. AIAA J 32: 1598–1605, 1994.

[25] Jaccard P: Étude comparative de la distribution florale dans une portion des Alpes et du Jura. Bulletin de la Société Vaudoise des Sciences Naturelles 37: 547, 1901.

[26] Wang S Tan J Yu Z: Study on the influence of dynamic/static interface processing methods on CFD simulation results of the axial-flow blood pump. Adv Mechan Eng 12: 168781402091057, 2020.

[27] Shannon CE: Communication in the presence of noise. Proc IRE 37: 10–21, 1949.

[28] Knüppel F Thomas I Wurm F-H Torner B: Suitability of different blood-analogous fluids in determining the pump characteristics of a ventricular assist device. Fluids 8: 151, 2023.

[29] Kameneva MV Burgreen GW Kono K Repko B Antaki JF Umezu M: Effects of turbulent stresses on mechanical hemolysis: Experimental and computational analysis. ASAIO Journal 50: 418–423, 2019 10.1097/01.mat.0000136512.36370.b5 PMC6400211 15497379

[30] Bortot M Ashworth K Sharifi A: Turbulent flow promotes cleavage of VWF (von Willebrand Factor) by ADAMTS13 (A Disintegrin and Metalloproteinase With a Thrombospondin Type-1 Motif, Member 13). Arterioscler Thromb Vasc Biol 39: 1831–1842, 2019. 31291760 10.1161/ATVBAHA.119.312814 PMC9109938

[31] Torner B Konnigk L Abroug N Wurm H: Turbulence and turbulent flow structures in a ventricular assist device-A numerical study using the large-eddy simulation. Int J Numer Method Biomed Eng 37: e3431, 2021. 33336869 10.1002/cnm.3431

[32] Konnigk L Torner B Bruschewski M Grundmann S Wurm F-H: Equivalent scalar stress formulation taking into account non-resolved turbulent scales. Cardiovasc Eng Technol 12: 251–272, 2021. 33675019 10.1007/s13239-021-00526-x PMC8169507

