# Supplementary material for: Numerical Analyses of Blood Pumps Under Realistic Operating Conditions
Source: ASAIO J. 2025 Nov 4;72(6):477–85. doi: 10.1097/MAT.0000000000002590 (PMC13218585; doi:10.1097/MAT.0000000000002590)
Supplement: Supplementary file 1 [file mat-72-477-s001.pdf]

Supplementary Materials

Waveform of the applied reservoir pressures and corresponding flow rate

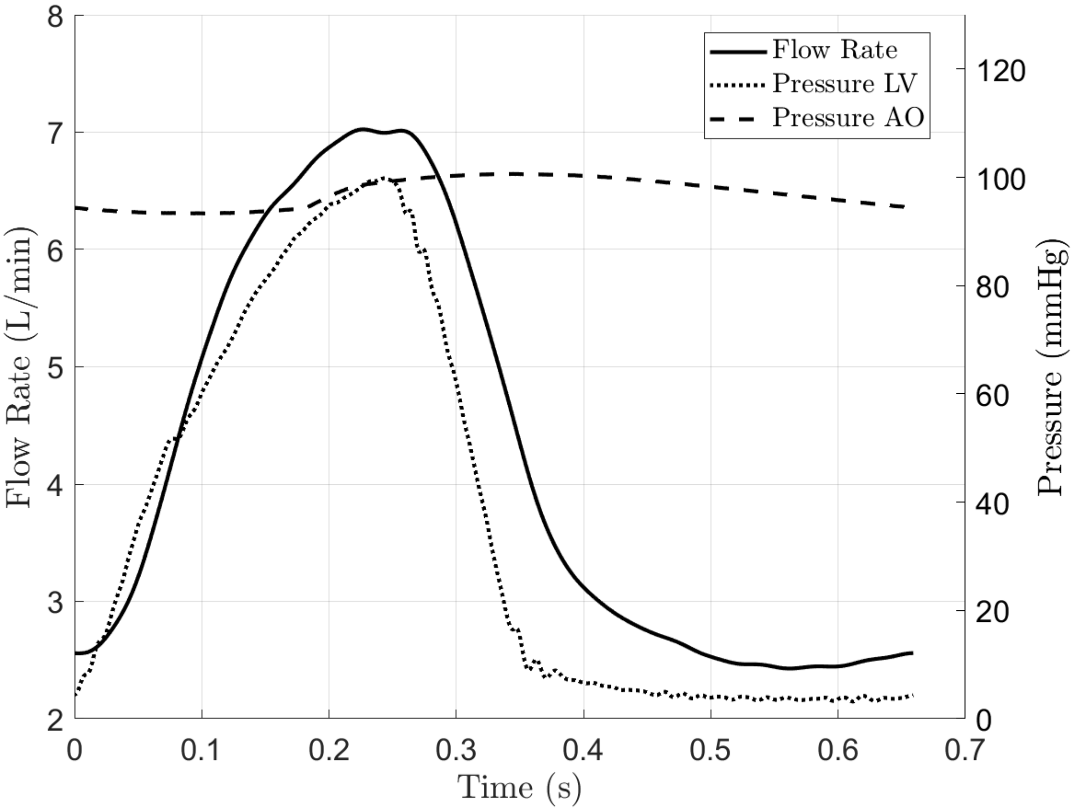

Figure 1: Time-resolved waveforms of the measured left ventricular and aortic pressures applied in the specific reservoir, along with the corresponding mass flow rate. The applied pressures are obtained from a lumped parameter model.

Statistics of the Measured Data

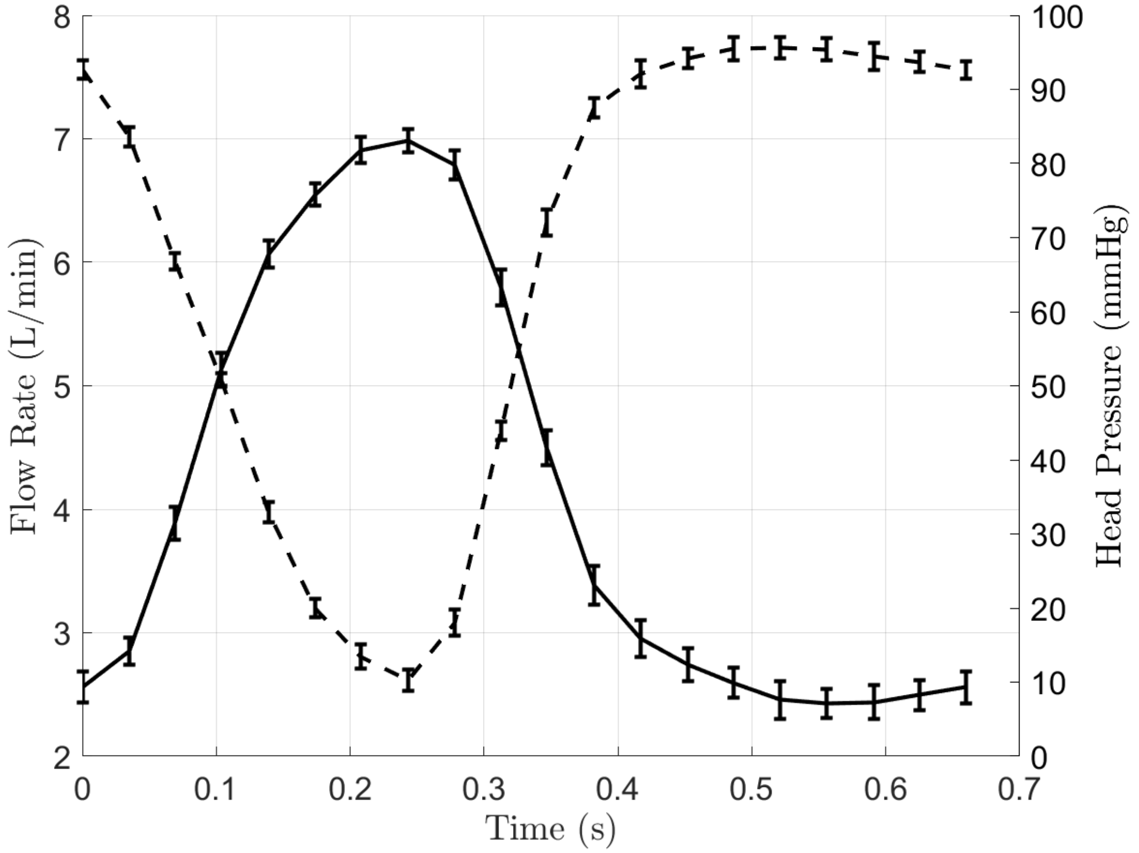

Figure 2: Mean value and standard deviation of measured pressure head and flow at 20 representative time points over 40 cardiac cycles.

Mesh Independence Study

The resulting error estimates of the mesh independence study were e^{21}_{a} = 1.78%, e^{21}_{exp} = 1.46%, and GCI^{21}_{fine} = 1.8%, with an apparent order of p = 3.65. The grid refinement ratios were r_{21} = 1.25 and r_{32} = 1.31.
